# Phenylketonuria Diagnosis by Massive Parallel Sequencing and Genotype-Phenotype Association in Brazilian Patients

**DOI:** 10.3390/genes12010020

**Published:** 2020-12-25

**Authors:** Rafael Hencke Tresbach, Fernanda Sperb-Ludwig, Rodrigo Ligabue-Braun, Tássia Tonon, Maria Teresinha de Oliveira Cardoso, Romina Soledad Heredia, Maria Teresa Alves da Silva Rosa, Bárbara Cátia Martins, Monique Oliveira Poubel, Luiz Carlos Santana da Silva, François Maillot, Ida Vanessa Doederlein Schwartz

**Affiliations:** 1 BRAIN Laboratory (Basic Research and Advanced Investigations in Neurosciences), Hospital de Clínicas de Porto Alegre, Porto Alegre, RS 90035-903, Brazil; tresbach@gmail.com (R.H.T.); tassitonon@gmail.com (T.T.); idadschwartz@gmail.com (I.V.D.S.); 2 Graduate Program in Genetics and Molecular Biology, Universidade Federal do Rio Grande do Sul, Porto Alegre, RS 91501-970, Brazil; 3 Graduate Program in Biological Sciences (PPGBio), Universidade Federal de Ciências da Saúde de Porto Alegre, Porto Alegre, RS 90050-170, Brazil; ligabue.braun@gmail.com; 4 Department of Pharmaceutical Sciences, Universidade Federal de Ciências da Saúde de Porto Alegre, Porto Alegre, RS 90050-170, Brazil; 5 Graduate Program in Medicine: Medical Sciences, Universidade Federal do Rio Grande do Sul, Porto Alegre, RS 90035-003, Brazil; 6 Serviço de Triagem Neonatal de Brasília, Brasília, DF 70684-831, Brazil; doencasraras.ses@gmail.com (M.T.d.O.C.); romina.rh@gmail.com (R.S.H.); mariateresagene@yahoo.com.br (M.T.A.d.S.R.); barbaracatiam@gmail.com (B.C.M.); monolivepoubel@gmail.com (M.O.P.); 7 Hospital de Apoio de Brasília, Unidade de Genética, Brasília, DF 70684-831, Brazil; 8 Faculdade de Medicina, Universidade Católica de Brasília (UCB), Brasília, DF 71966-700, Brazil; 9 Laboratory of Inborn Errors of Metabolism, Institute of Biological Sciences, Federal University of Pará, Belém, PA 66075-110, Brazil; lcss@ufpa.br; 10 CHRU et université de Tours, INSERM 1253 “iBrain”, 37032 Tours, France; francois.maillot@univ-tours.fr; 11 Medical Genetics Service, Hospital de Clínicas de Porto Alegre, Porto Alegre, RS 90035-903, Brazil; 12 Department of Genetics, Universidade Federal do Rio Grande do Sul (UFRGS), Porto Alegre, RS 91501-970, Brazil

**Keywords:** next-generation sequencing, molecular diagnosis, phenylketonuria, phenylalanine hydroxylase, *PAH*

## Abstract

Phenylketonuria (PKU) is a common inborn error of amino acid metabolism in which the enzyme phenylalanine hydroxylase, which converts phenylalanine to tyrosine, is functionally impaired due to pathogenic variants in the *PAH* gene. Thirty-four Brazilian patients with a biochemical diagnosis of PKU, from 33 unrelated families, were analyzed through next-generation sequencing in the Ion Torrent PGM™ platform. Phenotype–genotype correlations were made based on the BioPKU database. Three patients required additional Sanger sequencing analyses. Twenty-six different pathogenic variants were identified. The most frequent variants were c.1315+1G>A (*n* = 8/66), c.473G>A (*n* = 6/66), and c.1162G>A (*n* = 6/66). One novel variant, c.524C>G (p.Pro175Arg), was found in one allele and was predicted as likely pathogenic by the American College of Medical Genetics and Genomics (ACMG) criteria. The molecular modeling of p.Pro175Arg indicated that this substitution can affect monomers binding in the PAH tetramer, which could lead to a change in the stability and activity of this enzyme. Next-generation sequencing was a fast and effective method for diagnosing PKU and is useful for patient phenotype prediction and genetic counseling.

## 1. Introduction

Phenylketonuria (PKU, OMIM #261600) is an autosomal recessive inborn error of metabolism in which the conversion of phenylalanine (Phe) to tyrosine by the phenylalanine hydroxylase (EC 1.14.16.1) is defective, resulting in partial or total inactivity of the conversion due to biallelic variants in the *PAH* gene [1]. Untreated Phe accumulation leads to irreversible neurological effects, such as impaired cognitive development in children and seizures [2].

The treatment for PKU consists of Phe-free dietary management and supplementation with the Phe-free metabolic formula [3,4]. The use of sapropterin dihydrochloride may be also recommended for tetrahydrobiopterin (BH_4_)-responsive patients [5].

In Brazil, the public health system neonatal screening program performs biochemical screening for PKU by the detection of Phe in dried blood spots (DBS). If the results are abnormal, an additional blood sample is requested to confirm the diagnosis and begin treatment. The confirmatory test includes the measurement of blood Phe and tyrosine concentrations [6].

The *PAH* gene comprises 13 exons and 12 introns, resulting in a 452-residue protein. Worldwide, about 1188 variants in the *PAH* gene have been described in the PAHvdb (http://www.biopku.org) and about 1013 variants in the Human Gene Mutation Database (HGMD, http://www.hgmd.cf.ac.uk) [7]. The molecular investigation is sometimes the key to concluding the diagnosis of PKU and, consequently, assists in improving the treatment. The gold standard for gene variant detection in PKU patients is Sanger sequencing, which is costly and time-consuming [8]. Next-generation sequencing allows massive parallel deep-level sequencing, i.e., analyzing the entire exome or a targeted gene panel, which results in increased diagnostic sensitivity, faster sequencing and an inexpensive process [9]. *PAH* genotype data can be used for the prediction of BH4 responsiveness [9]. 

This study aimed to perform a molecular diagnosis of Brazilian PKU patients through massive parallel sequencing to confirm the diagnosis and obtain new data that can improve the choice of treatment for some patients.

## 2. Materials and Methods

### 2.1. Subjects

A total of 34 (33 nonrelated) nonconsanguineous PKU patients were recruited (female = 18, classic PKU = 22, mild PKU = 10, and undefined PKU type = 2), of whom 7 had complete previous genotyping, and 7 had incomplete previous genotyping. Of the total cohort, 23 patients were seen at the HCPA Medical Genetics Service (Porto Alegre, Rio Grande do Sul-RS, Brazil), and 11 were seen at the Hospital de Apoio de Brasília Neonatal Service on Newborn Screening, Genetics Unit (Distrito Federal-DF, Brazil). 

For the patients from RS, a BH_4_ deficiency was previously excluded by the measurement of 6,7-dihydropteridine reductase (DHPR) activity in the blood or DBS and of biopterins and neopterins in urine or DBS. Information such as the Phe level at diagnosis, age at diagnosis, age at treatment initiation, BH_4_ responsiveness [10,11], and previous genotyping diagnosis of the patients were obtained retrospectively from the medical records. 

### 2.2. DNA Extraction and Sequencing

Total blood samples were collected, and DNA extraction was performed with an Easy-DNA gDNA Purification Kit (Thermo Fisher Scientific, Waltham, MA, USA), according to the manufacturer’s instructions. The DNA samples were quantified in Qubit (Thermo Fisher Scientific).

A targeted gene panel was designed using the Ion AmpliSeq Designer (Thermo Fisher Scientific) to include all the exonic regions and intron–exon boundaries of the *PAH* gene and of the genes causing BH_4_ deficiencies (*GCH1*, *GCHFR*, *PTS*, *PCBD1*, *QDPR*, and *SPR*). Genomic DNA libraries were prepared using an Ion AmpliSeq™ Library Kit 2.0 (Thermo Fisher Scientific), followed by purification with magnetic beads (AMPure beads). The samples were sequenced in an Ion Torrent PGM Platform (Thermo Fisher Scientific, server version 5.0, Waltham, MA, USA), with a minimal coverage of 250X at the Unidade de Pesquisa Laboratorial (Centro de Pesquisa Experimental, Hospital de Clínicas de Porto Alegre).

Massive parallel sequencing data were analyzed using Torrent Suite 5.0.5 (Thermo Fisher Scientific) to perform the base-calling procedure. IGV 2.8.2 [12] was used for detection of the depth of sequencing and coverage failures that could suggest deletions. Variants were filtered by Enlis Genome Research (Enlis Genomics, Berkeley, CA, USA) and Ion Reporter software (Thermo Fisher Scientific), as well as the following databases: ClinVar, Phenylalanine Hydroxylase Gene Locus-Specific (*PAH*vdb) [9], and Human Gene Mutation Database.

Novel, conflicting and phase undetermined variants were confirmed by automated Sanger sequencing in an ABI 3500 Genetic Analyzer (Applied Biosystems, Foster City, CA, USA). The results were analyzed in Chromas 2.6.1 (Technelysium, South Brisbane, Australia), and NM_000277.3 and NP_000268.1 were used as the reference sequences.

Previous genotypes were identified through the Sanger sequencing method.

### 2.3. Pathogenicity Determination and Prediction 

To determine the pathogenicity of the novel variant, the following variables were considered: allele frequency < 1% in gnomAD [13] and ABraOM [14]. The American College of Medical Genetics and Genomics (ACMG) guidelines for interpreting variants were used [15].

### 2.4. Genotype–Phenotype Analysis

Genotype–phenotype associations were made through BioPKU database entries [16] and biochemical classification (classic, mild, or undefined PKU), as previously described by Nalin et al. [17], using as the main criteria the Phe level at diagnosis (classic: >1200 µMol/L and mild: 360–1200 µMol/L).

### 2.5. Molecular Modeling

The tridimensional structure of wild-type phenylalanine hydroxylase was taken from Protein Data Base (PDB) ID 6HYC [18], which also served as a template for tetramer reconstruction. The point mutations were modeled with DeepView [19], while the frameshift and early stop codon variants were modeled with I-TASSER [20]. FoldX 5.0 (AnalyseComplex command) was used to inspect the possible differences in binding affinity between monomers in the PAH tetramer. The differences between the energies of the mutant and wild-type proteins (ΔΔG = ΔGmut − ΔGwt) were considered significant above 1.6 kcal/mol. This value corresponds to twice the intrinsic standard deviation of FoldX [21] and should significantly affect the stability of the variant [22].

## 3. Results

The clinical, biochemical, and genotypic results are presented in Table 1. The sample’s median age at diagnosis was 37 [interquartile (IQ) 27–60] days. For the RS patients, the median age at diagnosis was 81.4 (IQ 26.5–88) days and 41 (IQ 34–45.5) days for the DF patients. 

**Figure 1:**
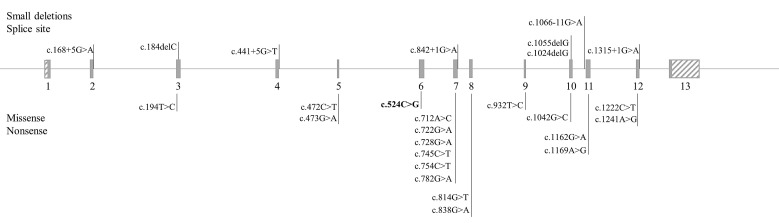
*PAH* exon structure, and the location of the variants found in the patient sample.

A total of 26 different pathogenic variants were found in the *PAH* gene (Table 2). One was a novel variant c.524C>G (p.Pro175Arg), five were located at the intron–exon boundaries, and twenty were found in exonic regions (Figure 1). The majority (*n* = 6) of the pathogenic variants were found in exon 7. For the other BH_4_ metabolism-related genes, no pathogenic variants were found. 

Of the 14 patients without a BH_4_ responsiveness test, the results of nine were predicted through the BioPKU database: two were responsive, and seven were nonresponsive. Of the total cohort, the results of ten agreed with the BioPKU data. Two RS patients (patients 2 and 7) presented a genotype not described in the BioPKU database [46] and were nonresponsive to BH_4_, according to the biochemical test [10,11]. Also, three DF patients (patients 26, 27 and 31) presented a genotype not described at BioPKU database, being two responsive and one nonresponsive, respectively.

The novel variant c.524C>G was found in patient 20, located on exon 6 of the *PAH*. The ACMG criteria fulfilled by the variant were PM2, PM5, PP2, and PP3, resulting in a likely pathogenic classification. In addition, the patient’s clinical information was consistent with classic PKU. 

**Figure 2:**
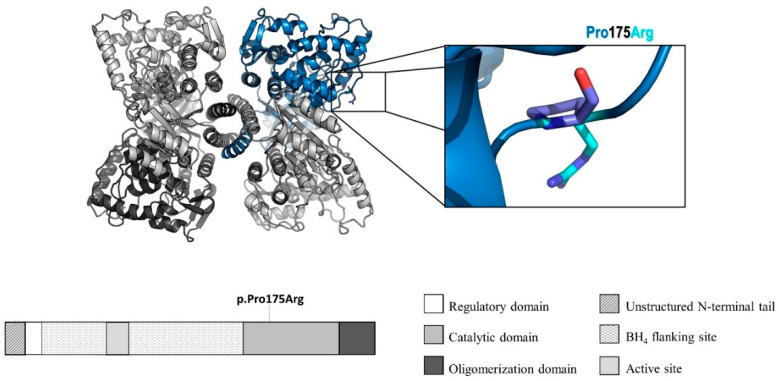
Molecular modeling and protein structure of the PAH enzyme, with the protein location of the novel variant p.Pro175Arg. Adapted from Flydal et al. [18].

As shown in Figure 2, the novel variant c.524C>G resulted in a proline being substituted with an arginine in position 175, which is located in the catalytic domain of the PAH protein. This variant does not promote structural alterations in the protein. In the combination of variants p.(Pro175Arg) and p.Arg252Trp, found in the genotype of patient 2, a small portion of monomers showed higher affinity between the subunits than the wild-type complex. The molecular modeling analysis of PAH variants p.Thr238Pro and p.Gly272Ter, found in patient 14, showed differences in the interaction energy between monomers in the PAH tetramer, and most of the different tetramers showed significantly lower affinity than the wild-type (Table S1).

## 4. Discussion

PKU is the most common IEM, and its incidence ranges between 1:850–112,000 in Europe (Karachay-Cherkessia Republic (Russia) and Finland, respectively], to 1:10,000 live births in the USA [47]. In Brazil, its incidence is 1:25,000 live births [48], while, in Southern Brazil, its incidence is 1:12,000–16,000 [49]. PKU has been included in Brazil’s newborn screening program since 2001 [50]. Despite this screening program, our sample’s median age at diagnosis was higher than the Brazilian Ministry of Health recommendations, i.e., up to 28 days of age [6]. A reason for this high median age at diagnosis is the difficulties in the execution of the program, which was implemented only in 2001. Some of our patients were born before that, when each Brazilian state performed a different screening and not all states included PKU in their newborn screening program. This is the reason behind the outstandingly late diagnosis of patients 22.1 and 22.2, diagnosed only after the development of severe symptoms. Besides that, this family is very interesting, since the oldest brother (22.2), who was diagnosed after—and because of—the youngest brother, presented a milder neurological phenotype.

The *PAH* gene analysis by massive parallel sequencing is a fast, cost-effective, and accurate alternative for the genetic diagnosis of PKU [8,51]. Due to its large size and heterogeneity, similar symptoms are caused by alterations in more than one gene, as in the differential diagnosis of BH_4_ deficiency and *DNAJC12* gene variants. In PKU, especially, a less time-consuming diagnosis can be helpful to avoid the development of neurological symptoms to help predict BH_4_ responsiveness and to facilitate a differential diagnosis [52].

In this study, the patients’ molecular diagnosis agreed in every case with the diagnosis based on biochemical and clinical observations, which confirms the effectiveness of this methodology. We identified variants that were not covered in the previous genotyping analysis. In patient 9, for example, the error in previous genotyping could have been the result of a lack of specificity or coverage of the implemented technique or a lack of analysis of the parents’ genotypes.

The most frequent variant found in the patients, c.1315+1G>A, was described as a common pathogenic variant in different Northern European populations, especially in Germany [53]. The second-most frequent variant, p.Arg158Gln, is also common in European populations, including Southern Italy and Eastern Europe [53]. The third-most prevalent variant found, p.Val388Met, is described as common in the Iberian Peninsula (5.7% in Spain and 19% in Portugal) and has a high frequency in Brazil (9%) and Chile (13%) [54]. 

The second-most frequent variant in RS patients, p.Arg408Trp, is also common in German populations (24.6%) [55]. In Southern Brazil, the predominance of European ancestry (77.7%) can explain these findings [56]. A previous study of the RS population found p.Ile65Thr (19.5%), c.169-13T>G (9.7%), p.Arg261Ter (9.7%), p.Arg261Gln (9.7%), p.Val388Met (9.7%), and p.Arg408Trp (9.7%) to be the most frequent variants in this population [23]. However, the frequency of these variations differed in the present study: p.Ile65Thr (2.1%), p.Arg261Gln (4.3%), p.Val388Met (8.7%), p.Arg408Trp (13%), and the variants c.169-13T>G and p.Arg261Ter were not found. Nevertheless, the small sample size in the previous study should be taken into consideration (*n* = 16). The variants p.Arg261Gln and c.1066-11G>A, frequent in patients from DF, have also been described as the most common variants in Portugal [53]. In the DF, which is in the Midwestern region of Brazil, the population’s ancestry shows a mixture of Southeastern and Northeastern Brazilian populations, with significant European (63%) and African (24.1%) ancestries [57].

A previous study by Acosta et al. (2001) [58] in a Brazilian population (a mixture of Southern, Southeastern, Northeastern, and Midwestern regions) described the most frequent of the pathogenic variants as c.1066-11G>A (17.4%), p.Arg261Gln (12.2%), p.Val388Met (9.1%), p.Arg252Trp (6.5%), and p.Arg270Lys (4.8%) [58]. Of these variants, only p.Arg270Lys was not found in the present study. The variants p.Arg261Gln, p.Val388Met, and c.1066-11G>A are also frequent in the States of Mato Grosso do Sul and Minas Gerais [59,60,61]. The most common pathogenic variants in Argentina and Chile were p.Arg261Gln (10.6%) and p.Val388Met (17.2%), respectively [62,63].

The novel variant p.Pro175Arg involves the substitution of a proline for an arginine. The hydrophobic amino acid proline has particular properties: its side chain is connected to the protein backbone. However, unlike proline, which does not display main-chain conformation, arginine, a charged amino acid, is usually found in protein-active or protein-binding sites [64]. The variant is located in the catalytic domain, although not in a hotspot region with highly destabilizing pathogenic variants between residues 238–330 [18]. The molecular modeling analysis indicates that this substitution can affect the binding between monomers in the PAH tetramer, which could lead to a change in the stability and activity of this enzyme. Another variant, p.Arg252Trp, has 1% of the PAH activity [65] and is related to the classic PKU.

## 5. Conclusions

The correlation of many variations in the genotypes and their resulting phenotypes is already available in public databases. Thus, a fast genotype diagnosis of PKU patients can help with treatment outcomes. Genotyping is a helpful way to understand how phenylalanine hydroxylase is altered in a patient, the impact of this specific alteration to the enzyme, and the enzyme’s level of residual activity with these variants. Additionally, genotyping can help with the patients whose genotypes have information of the BH_4_ responsiveness; when these patients are responsive, the supplementation with BH_4_ leads to the enhancement of residual PAH activity, with a chaperone-like effect on a misfolding enzyme subunit [66].

This study presents a summary of the clinical and genetic data of 33 unrelated patients from two different regions of Brazil, which confirmed the diagnosis of PAH deficiency in every case. A novel variant was found in the *PAH* gene.

## Figures and Tables

**Table 1:** Summary of the included phenylketonuria (PKU) patients, including genotypes and clinical information.

Patient	State	Gender	First Phe Level (µMol/L)	Age at Diagnosis (Days)	Age at Treatment Starting (Days)	Type of PKU	NGS Genotype	Previous Genotype	Type of PKU According to BioPKU ^1^	BH4 Responsiveness According to the Test	BH4 Responsiveness According to BioPKU ^2^
1 *	RS	F	1566	26	26	C	c.1222C>T(;)1222C>T p.Arg408Trp(;)Arg408Trp	c.1222C>T(;)1222C>T p.Arg408Trp(;)Arg408Trp	Classic (1832/1845)	NP	No (95/98) Yes (2/98) Slow (1/98)
2 **	RS	M	847	28	58	M	c.**524C**>**G**(;)754C>T p.(**Pro175Arg**)(;)Arg252Trp	c.754C>T(;)? p.Arg252Trp(;)?	NA	No *****	NA
3	RS	F	1784	36	36	C	c.1042C>G(;)1315+1G>A p.Leu348Val(;)?	c.1042C>G(;)1315+1G>A p.Leu348Val(;)?	Classic (11/16)	NP	No (5/6)Yes (1/6)
4	RS	F	1478	43	43	C	c.932T>C(;)1315+1G>A p.Leu311Pro(;)?	c.1315+1G>A(;)? p.?(;)?	Classic (2/2)	NP	NA
5	RS	M	417	28	50	M	c.1162G>A(;)1169A>G p.Val388Met(;)Glu390Gly	c.1162G>T(;)? p.Val388Met(;)?	Mild (8/12)	NP	Yes (11/11)
6	RS	F	375	32	66	M	c.1066-11G>A(;)1169A>G p.Gln355_Tyr356insGlyLeuGln(;)Glu390Gly	NP	Mild (8/14)	NP	Yes (8/8)
7 **	RS	M	NA	60	60	U	c.842+1G>A(;)1162G>A p.(?)(;)Val388Met	c.842+1G>A(;)1162G>Ap.?(;)Val388Met	NA	No *****	NA
8	RS	F	562	74	82	M	c.1169A>G(;)1222C>T p.Glu390Gly(;)Arg408Trp	NP	Mild (54/84)	Yes	Yes (23/23)
9	RS	M	1845	102	102	M	c.722G>A(;)1222C>T p.Arg241His(;)Arg408Trp	c.1222C>T(;)1222C>T p.Arg408Trp(;)Arg408Trp	Mild (25/28)	No ******	Yes (3/6) Slow (2/6) No (1/6)
10 *	RS	M	877	128	156	M	c.473G>A(;)1162G>A p.Arg158Gln(;)Val388Met	c.1162G>A(;)? p.Val388Met(;)?	Classic (5/7)	No	Yes (2/3) Slow (1/3)
11 ***	RS	M	1022	195	292	M	c.[1241A>G];[1042C>G] p.[Leu348Val];[Tyr414Cys]	NP	Mild (4/5)	Yes *****	Yes (3/3)
12	RS	F	3245	5	44	C	c.745C>T(;)838G>A p.Leu249Phe(;)Glu280Lys	NP	Classic (1/1)	NP	NA
13	RS	F	1361	15	19	C	c.754C>T(;)1222C>T p.Arg252Trp(;)Arg408Trp	c.1222C>T(;)? p.Arg408Trp(;)?	Classic (103/103)	NP	No (4/4)
14 ***	RS	F	1736	16	16	C	c.[473G>A];[1055delG] p.[Arg158Gln];[Gly352ValfsTer48]	NP	Classic (1/1)	NP	NA
15	RS	F	2329	24	24	C	c.712A>C(;)814G>T p.Thr238Pro(;)Gly272Ter	NP	Classic (1/1)	NP	NA
16	RS	M	2716	27	27	C	c.194T>C(;)472C>T p.Ile65Thr(;)Arg158Trp	c.194T>C(;)? p.Ile65Thr(;)?	Classic (2/2)	NP	No (1/1)
17	RS	M	1697	30	30	C	c.754C>T(;)1024delG p.Arg252Trp(;)Ala342HisfsTer58	NP	NA	NP	NA
18 **	RS	F	2178	27	48	C	c.754C>T(;)1315+1G>A p.Arg242Trp(;)?	c.1315+1G>A(;)? p.?(;)?	Classic (9/9)	NP	No (1/1)
19	RS	M	2904	73	101	M	c.473G>A(;)1162G>A p.Arg158Gln(;)Val388Met	NP	Classic (5/7)	NP	No (2/3) Slow (1/3)
20	RS	M	2323	4	17	C	c.1222C>T(;)1315+1G>A p.Arg408Trp(;)?	c.1222C>T(;)1315+1G>A p.Arg408Trp(;)?	Classic (265/265)	NP	No (40/40)
21	RS	M	2081	227	233	C	c.473G>A(;)1315+1G>A p.Arg158Gln	NP	Classic (29/29)	No *****	No (6/6)
22.1 ****	RS	M	1455	670	670	C	c.782G>A(;)1315+1G>A p.Arg261Gln(;)?	c.782G>A(;)1315+1G>A p.Arg261Gln(;)?	Classic (47/66)	No *****	No (24/25) Slow (1/25)
22.2 ****	RS	M	2196	2555	2677	C	c.782G>A(;)1315+1G>A p.Arg261Gln(;)?	c.782G>A(;)1315+1G>A p.Arg261Gln(;)?	Classic (47/66)	No *****	No (24/25) Slow (1/25)
23	DF	M	1978	39	39	C	c.754C>T(;)1066-11G>A p.Arg252Trp(;) Gln355_Tyr356insGlyLeuGln	NP	Classic (19/19)	No	No (6/6)
24	DF	M	1857	18	22	C	c.168+5G>A(;)782G>A p.?(;)Arg261Gln	NP	Mild (4/5)	Slow	Yes (2/2)
25	DF	M	768	47	47	M	c.184delC(;)1169A>G p.Leu62Ter(;)Glu390Gly	NP	NA	Yes	NA
26	DF	F	344	41	41	M	c.184delC(;)1169A>G p.Leu62Ter(;)Glu390Gly	NP	NA	Yes	NA
27	DF	F	3133	38	38	C	c.1066-11G>A(;)1066-11G>A p.Gln355_Tyr356insGlyLeuGln(;)Gln355_Tyr356insGlyLeuGln	NP	Classic (420/427)	Yes	No (107/114) Slow (7/114)
28	DF	F	1724	41	41	C	c.728G>A(;)728G>A p.Arg243Gln(;)Arg243Gln	NP	Classic (140/141)	No	No (13/14) Slow (1/14)
29	DF	F	1936	44	44	C	c.441+5G>T(;)473G>A p.Arg158Gln(;)?	NP	Classic (20/21)	No	No (9/12) Slow (3/12)
30	DF	F	2299	22	22	C	c.184delC(;)184delC p.Leu62Ter(;)Leu62Ter	NP	NA	No	NA
31	DF	F	1754	57	57	C	c.473G>A(;)782G>A p.Arg158Gln(;)Arg261Gln	NP	Mild (21/36)	Yes	Yes (8/13) No (4/13) Slow (1/13)
32	DF	F	NA	60	NA	U	c.1162G>A(;)1162G>A p.Val388Met(;)Val388Met	NP	Classic (23/41)	Yes	Yes (9/15) No (4/15) Slow (2/15
33	DF	F	1361	30	30	C	c.782G>A(;)1315+1G>A p.Arg261Gln(;)?	NP	Classic (47/66)	Yes	No (24/25) Slow (1/25)

Notes: In bold: novel variant, NP: not performed, NA—not available, C: classic, M: mild, and U: undefined. ^1^—The most frequent type in the BioPKU database. ^2^—The most frequent responsiveness phenotype informed of in the BioPKU. *—This patient had genotype validation by Sanger sequencing; ** These patients were previously described in [23]. *** Allelic phase confirmed by parents’ analysis. **** These patients are siblings. ***** The BH4 responsiveness results were described by [10]. ****** The BH4 responsiveness results were described by [11]. NGS: next-generation sequencing.

**Table 2:** Variants found in 33 unrelated PKU patients, their references, and American College of Medical Genetics and Genomics (ACMG) classification.

Allele	Protein	Location	Reference	ACMG	Effect
c.168+5G>A	p.?	I 2	[24]	PP5	VUS
c.184delC	p.Leu62Ter	E 3	[25]	PVS1, PM2, PP3, PM4	Pathogenic
c.194T>C	p.Ile65Thr	E 3	[26]	PS3, PP2, PP5	Likely pathogenic
c.441+5G>T	p.?	I 4	[24]	PP5	VUS
c.472C>T	p.Arg158Trp	E 5	[27]	PS1, PP2, PP3, PP5	Likely pathogenic
c.473G>A	p.Arg158Gln	E 5	[28]	PS1, PP2, PP3, PP5	Likely pathogenic
c.524C>G	p.(Pro175Arg)	E 6	This article	PM2, PM5, PP2, PP3	Likely pathogenic
c.712A>C	p.Thr238Pro	E 7	[29]	PM2, PP2, PP3, PP5	VUS
c.722G>A	p.Arg241His	E 7	[30]	PS1, PS3, PP2, PP3, PP5	Pathogenic
c.728G>A	p.Arg243Gln	E 7	[31]	PS1, PS3, PP2, PP3, PP5	Pathogenic
c.745C>T	p.Leu249Phe	E 7	[32]	PS1, PP2, PP3, PP5	Likely pathogenic
c.754C>T	p.Arg252Trp	E 7	[33]	PS1, PS3, PP2, PP3, PP5	Pathogenic
c.782G>A	p.Arg261Gln	E 7	[33]	PS1, PS3, PP2, PP3, PP5	Pathogenic
c.814G>T	p.Gly272Ter	E 7	[34]	PVS1, PM4, PP3, PP5	Pathogenic
c.838G>A	p.Glu280Lys	E 7	[35]	PS1, PS3, PP2, PP3, PP5	Pathogenic
c.842+1G>A	p.?	I 7	[36]	PVS1, PP5	VUS
c.932T>C	p.Leu311Pro	E 9	[37]	PS1, PS3, PP2, PP3, PP5	Pathogenic
c.1024delG	p.Ala342HisfsTer58	E 10	[38]	PVS1, PM2, PM4, PP3, PP5	Pathogenic
c.1042C>G	p.Leu348Val	E 10	[26]	PS3, PP2, PP3, PP5	Likely pathogenic
c.1055delG	p.Gly352ValfsTer48	E 10	[39]	PVS1, PM4, PP3, PP5	Pathogenic
c.1066-11G>A	p.Gln355_Tyr356insGlyLeuGln	I 10	[40]	PS3, PP5	VUS
c.1162G>A	p.Val388Met	E11	[41]	PS1, PS3, PP2, PP3, PP5	Pathogenic
c.1169A>G	p.Glu390Gly	E 11	[42]	PS3, PS1, PP2, PP3, PP5	Pathogenic
c.1222C>T	p.Arg408Trp	E 12	[43]	PS3, PP2, PP3, PP5	Likely pathogenic
c.1241A>G	p.Tyr414Cys	E 12	[44]	PS1, PS3, PP2, PP3, PP5	Pathogenic
c.1315+1G>A	p.?	I 12	[45]	PVS1, PP5	VUS

Notes: E: exon and I: intron. The most frequent variant was c.1315+1G>A (8/66, 11.7%), followed by c.473G>A (6/66, 8.8%) and c.1162G>A (6/66, 8.8%). In the RS patients, the most common variants were c.1315+1G>A (7/44, 15.2%), c.1222C>T (6/44, 13%), c.473G>A (4/44, 8.7%), c.754C>T (4/44, 8.7%), and c.1162G>A (4/44, 8.7%). In the DF patients, c.184delC (4/22, 18.1%), c.782G>A (3/22, 13.6%), and c.1066-11G>A (3/22, 13.6%) were the most common variants.

## Supplementary Materials

The following are available online at https://www.mdpi.com/2073-4425/12/1/20/s1, Table S1. Differences in binding affinity between monomers in the PAH tetramer in patients 2 and 14 as predicted by the program FoldX 5.0. Interaction energy (ΔG) between monomers A to D calculated using combinations of the allelles 1 and 2 found in each patient. Differences between the energies of mutant and wild-type proteins (ΔΔG = ΔGmut−ΔGwt) above 1.6 kcal/mol should significantly affect the stability of the tetramer.
