# Supplementary material for: Phenylketonuria Diagnosis by Massive Parallel Sequencing and Genotype-Phenotype Association in Brazilian Patients
Source: Genes (Basel). 2020 Dec 25;12(1):20. doi: 10.3390/genes12010020 (PMC7824641; doi:10.3390/genes12010020)
Supplement: Supplementary file 1 [file genes-12-00020-s001.pdf]

Scheme 1. Differences in binding affinity between monomers in the PAH tetramer in patients 2 and 14 as predicted by the program FoldX 5.0. Interaction energy (ΔG) between monomers A to D calculated using combinations of the allelles 1 and 2 found in each patient. Differences between the energies of mutant and wild−type proteins (ΔΔG = ΔGmut − ΔGwt) above 1.6 kcal/mol should significantly affect the stability of the tetramer.

Energies data.							ΔΔG = ΔGmut−ΔGwt..						
**Patient 2.**	p.Pro175Arg(;)Arg252Trp..												
	**AB**	**AC**	**AD**	**BC**	**BD**	**CD**		**AB**	**AC**	**AD**	**BC**	**BD**	**CD**
**1222**	−46.523	−8.438	−0,343	−0,195	−5.842	−42.918	**1222**	−0,539	−0,974	−0,042	−0,029	−0,408	−1.357
**2122**	−48.010	−8.624	−0,315	−0,232	−6.116	−44.107	**2122**	−2.026	−1.161	−0,014	−0,065	−0,682	−2.547
**2212**	−46.876	−8.592	−0,348	−0,212	−5.923	−42.796	**2212**	−0,893	−1.129	−0,047	−0,045	−0,489	−1.236
**2221**	−47.380	−8.333	−0,338	−0,237	−5.774	−43.346	**2221**	−1.396	−0,87	−0,038	−0,07	−0,34	−1.786
**1122**	−46.739	−8.440	−0,344	−0,21	−5.838	−42.988	**1122**	−0,755	−0,977	−0,043	−0,043	−0,404	−1.427
**1212**	−46.614	−8.427	−0,342	−0,219	−5.894	−42.710	**1212**	−0,63	−0,963	−0,042	−0,053	−0,46	−1.150
**1221**	−46.645	−7.871	−0,341	−0,178	−6.111	−43.995	**1221**	−0,661	−0,408	−0,04	−0,012	−0,677	−2.434
**2112**	−47.519	−8.780	−0,362	−0,239	−6.136	−43.197	**2112**	−1.535	−1.317	−0,062	−0,073	−0,702	−1.636
**2121**	−48.936	−7.985	−0,367	−0,235	−6.210	−43.796	**2121**	−2.953	−0,521	−0,066	−0,069	−0,776	−2.235
**2211**	−48.755	−8.658	−0,348	−0,202	−6.321	−42.816	**2211**	−2.772	−1.194	−0,047	−0,035	−0,887	−1.256
**1112**	−46.739	−8.443	−0,321	−0,21	−6.126	−42.685	**1112**	−0,755	−0,979	−0,02	−0,043	−0,693	−1.124
**1121**	−46.739	−8.333	−0,341	−0,192	−5.766	−44.178	**1121**	−0,755	−0,869	−0,04	−0,026	−0,332	−2.617
**2111**	−47.738	−8.610	−0,339	−0,239	−5.926	−43.069	**2111**	−1.755	−1.147	−0,038	−0,073	−0,492	−1.509
**1211**	−46.885	−8.432	−0,351	−0,23	−5.903	−42.991	**1211**	−0,901	−0,968	−0,05	−0,064	−0,469	−1.431
**1111**	−46.739	−8.441	−0,344	−0,21	−5.889	−42.979	**1111**	−0,755	−0,977	−0,043	−0,043	−0,455	−1.419
**2222**	−47.882	−8.129	−0,344	−0,215	−5.888	−43.082	**2222**	−1.899	−0,665	−0,043	−0,048	−0,454	−1.521
**WT**	−45.984	−7.464	−0,301	−0,166	−5.434	−41.560							
**Patient 14**	p.Arg158Gln(;)Gly352ValfsTer48												
	**AB**	**AC**	**AD**	**BC**	**BD**	**CD**		**AB**	**AC**	**AD**	**BC**	**BD**	**CD**
**1222**	−13.504	−0,31	−0,036	0,006	−0,092	−4.755	**1222**	32.480	7.154	0,265	0,172	5.341	36.805
**2122**	−2.238	−0,531	0,001	−0,04	−0,038	−4.418	**2122**	43.746	6.933	0,302	0,126	5.396	37.142
**2212**	−4.133	0,09	0,004	−0,021	−0,111	−14.279	**2212**	41.851	7.554	0,305	0,145	5.322	27.281
**2221**	−4.133	−0,4	−0,038	0,001	−0,082	−14.457	**2221**	41.850	7.063	0,263	0,167	5.352	27.103

**1122**	−48.328	0,103	−0,012	−0,076	−0,024	−4.134	**1122**	−2.345	7.567	0,289	0,09	5.410	37.426
**1212**	−13.565	−8.426	−0,03	−0,072	−0,085	−14.433	**1212**	32.419	−0,962	0,271	0,094	5.348	27.128
**1221**	−13.613	−0,033	−0,552	0,004	−0,059	−7.001	**1221**	32.371	7.430	−0,251	0,171	5.375	34.560
**2112**	−3.617	0,014	0,001	−0,588	−0,092	−15.995	**2112**	42.367	7.477	0,302	−0,421	5.342	25.566
**2121**	−5.948	−0,49	−0,08	−0,066	−6.369	−5.050	**2121**	40.035	6.973	0,221	0,1	−0,935	36.511
**2211**	−4.133	0,367	−0,078	−0,012	0,028	−42.931	**2211**	41.851	7.830	0,223	0,155	5.461	−1.370
**1112**	−47.619	−8.370	−0,052	−0,282	−0,04	−13.244	**1112**	−1.635	−0,906	0,249	−0,116	5.394	28.316
**1121**	−46.868	0,105	−0,717	−0,083	−6.316	−4.620	**1121**	−0,884	7.568	−0,416	0,083	−0,882	36.940
**2111**	−8.452	−0,42	−0,083	−0,485	−6.255	−44.368	**2111**	37.532	7.044	0,218	−0,319	−0,821	−2.807
**1211**	−13.906	−8.124	−0,111	−0,096	−0,017	−43.545	**1211**	32.077	−0,661	0,19	0,07	5.417	−1.985
**1111**	−46.027	−8.071	−0,343	−0,236	−5.897	−44.191	**1111**	−0,043	−0,608	−0,042	−0,07	−0,463	−2.630
**2222**	−4.133	−0,35	−0,001	−0,001	−0,08	−3.595	**2222**	41.850	7.113	0,3	0,166	5.354	37.965
**WT**	−45.984	−7.464	−0,301	−0,166	−5.434	−41.560							

Note: Differences above 1.6 kcal/mol are considered significant. Differences above −1.6 kcal/mol suggests more affinity between subunits than the wild−type. Differences above +1.6 kcal/mol suggests less afinity between subunits than the WT.
